# Psychological Well-Being in Adults Across the COVID-19 Pandemic: A Two-Year Longitudinal Study

**DOI:** 10.3389/ijph.2025.1608347

**Published:** 2025-07-25

**Authors:** Melanie Ehrler, Alexandra Vogt, Dominique Eichelberger, Matthias Greutmann, Cornelia F. Hagmann, Oskar G. Jenni, Oliver Kretschmar, Markus A. Landolt, Beatrice Latal, Flavia M. Wehrle

**Affiliations:** ^1^ Child Development Center, University Children’s Hospital Zurich, Zurich, Switzerland; ^2^ Children’s Research Center, University Children’s Hospital Zurich, Zurich, Switzerland; ^3^ Institute of Psychiatry, Psychology and Neuroscience, King’s College London, London, United Kingdom; ^4^ Department of Psychosomatics and Psychiatry, University Children’s Hospital Zurich, Zurich, Switzerland; ^5^ Clinic for Cardiology, University Hospital Zurich, Zurich, Switzerland; ^6^ Department of Neonatology and Intensive Care, University Children’s Hospital Zurich, Zurich, Switzerland; ^7^ University of Zurich, Zurich, Switzerland; ^8^ Department of Cardiology, University Children’s Hospital Zurich, Zurich, Switzerland; ^9^ Division of Child and Adolescent Health Psychology, Department of Psychology, University of Zurich, Zurich, Switzerland; ^10^ University Research Priority Program (URPP), Adaptive Brain Circuits in Development and Learning (AdaBD), University of Zurich, Zurich, Switzerland

**Keywords:** psychological well-being, COVID-19, adults, longitudinal study, quality of life

## Abstract

**Objectives:**

The COVID-19 pandemic significantly affected global psychological well-being. We explored long-term trajectories of adults’ well-being from before the pandemic through its progression and identified risk factors for compromised well-being.

**Methods:**

Psychological well-being of a diverse group of 481 adults (18–74 years) was assessed prior to (T0) and during the pandemic (T1-T5: 04-05/2020, 10-11/2020, 04-05/2021, 10-11/2021, 04-05/2022). Latent variable mixture modelling identified subgroups with distinct trajectories of well-being. Ordinal regression analysis investigated risk factors for low well-being during the pandemic.

**Results:**

Three subgroups with different trajectories were identified: 73% reported consistently good well-being; 21% experienced decreasing well-being; and 5% exhibited consistently low well-being. Decreasing or consistently low well-being was significantly associated with younger age, limited social support, caregiving responsibilities, concerns about COVID-19 infection, and stress due to pandemic-related changes.

**Conclusion:**

While many individuals remained resilient, a vulnerable subgroup experienced mental health challenges over 2 years of the pandemic. Given the global scale, even a small affected proportion represents millions of people. Public health measures are essential to identify and support those at highest risk for impaired psychological well-being.

## Introduction

Already in the very early stages of the COVID-19 pandemic, concerns were voiced about its potential short- and long-term effects on the psychological well-being of the population (e.g., [1]). Countless studies have since been published to investigate this issue. Meta-analytic evidence, indeed, demonstrates a small but significant initial increase of symptoms of anxiety and depression, and a decrease of general mental health and psychological well-being compared to before the outbreak of the pandemic [2–4]. In contrast, the findings on the longer-term effects are much less consistent: While some studies found a return of psychological well-being to pre-pandemic levels within a few weeks or months after the outbreak (e.g., [5–8]), others reported persistent impairments, even beyond the first year of the pandemic (e.g., [9–12]).

It has been noted that solely investigating changes in group means of mental health symptoms and psychological well-being (i.e., group-level analyses) may mask the large inter-individual variability between individuals (e.g., [4]). Accordingly, latent variable mixture modelling can help investigating individual trajectories (i.e., individual-level analysis) to identify subgroups with distinct patterns of change. For instance, a study that investigated mental health trajectories from before the outbreak of the pandemic and across multiple time-points from April to October 2020 identified several distinct patterns, including a majority group with consistently good well-being alongside smaller groups experiencing either consistently impaired well-being, recovery after an initial drop or a deterioration across time [6]. Several other studies have described similar trajectories of psychological well-being in the general population as the pandemic continued to evolve over 2020 and 2021, however, these studies did not consider pre-pandemic well-being [13–17].

To date, there is a lack of studies investigating distinct trajectories of psychological well-being beyond mid-2021. However, this is important for a comprehensive understanding of changes across the pandemic as the population’s general mental health and well-being has been shown to covary with the number of COVID-19 cases, COVID-19-related deaths and the stringency of governmental measures to prevent the spread of the virus (e.g., [4, 15, 18, 19]) – all of which continued to evolve well beyond the first year of the pandemic [20].

Thus, the current study aims to expand previous research by i) describing different trajectories of psychological well-being in a heterogeneous group of adults from prior to the outbreak of the COVID-19 pandemic and over the course of 2 years (i.e., until mid-2022) and by ii) identifying demographic, social and pandemic-related factors that predict an individual’s psychological well-being over time.

## Methods

### Study Design and Sample

The sample of the current study was composed of five cohort studies at the University Children’s Hospital Zurich, Switzerland. In all of them, psychological well-being had been assessed prior to the outbreak of the COVID-19 pandemic. Study 1 [21] investigated young adults with congenital heart disease (CHD) who were treated at the cardiac outpatient clinic of the University Hospital Zurich, Switzerland. Patients were between 18 and 30 years of age and without severe neurological impairments or genetic syndromes. Healthy peers were recruited into a control group. Study 2 [22] followed very preterm born children (VPT; <32 weeks of gestation) and their parents from birth to school age. Typically developing term-born peers and their parents were recruited into a control group. Parents were between 33 and 56 years of age at the latest follow up. Study 3 [23] followed children with congenital heart disease (CHD) and their parents from their first cardiopulmonary bypass surgery until school age. Parents were between 30 and 74 years of age at the latest follow up. Of study 2 and 3, only parents were included in the current analyses. For the findings on child well-being during the COVID-19 pandemic, see [10, 24]. Study 4 [25] followed a non-clinical sample of individuals born between 1954 and 1961. At the latest follow-up, they were between 58 and 66 years old. Study 5 [25] followed a non-clinical sample of individuals born between 1974 and 1979, either at term or preterm (<37 weeks of gestation). At the latest follow-up, they were between 40 and 45 years old. Data assessments of the five studies took place between 2014 and mid-March 2020. For the detailed recruitment procedure and sample size of each study, please refer to Supplementary Figure S1. For the current study, participants were eligible if they had completed a set of questionnaires on their psychological well-being as part of one of these studies prior to the outbreak of the COVID-19 pandemic (T0).

Eligible participants were approached again during the first wave of the pandemic while lockdown measures were in place in Switzerland (T1: April/May, 2020). They completed an online survey to report on their psychological well-being and on how the pandemic impacted their live. Participants who completed the T1 assessment were approached again every 6 months until a final assessment in spring 2022 (T2: October/November, 2020; T3: April/May, 2021; T4: October/November, 2021; T5: April/May, 2022). Supplementary Table S1 lists the restriction measures in Switzerland at each assessment time-point. The ethics committee of the Canton of Zurich approved each individual study and additional data collection during the pandemic in accordance with the declaration of Helsinki. All participants gave written informed consent.

### Assessment Instruments

To assess psychological well-being, the *mental* subscale of the 12-item version of the *Short Form Health* questionnaire (SF-12) was used [26, 27]. This scale had been assessed in all five studies prior to the pandemic (T0) and was assessed as part of an online survey at T1, T2, T3, T4 and T5. The mental subscale of the SF-12 assesses four dimensions: *vitality*, *social function*, *role limitations due to emotional problems* and *mental health*. Raw scores were transformed into T-values based on German norms (n = 2524; [27]). The SF-12 mental subscale has good internal consistency (Cronbach’s α = 0.89) [27]. Higher scores indicate better psychological well-being.

Several demographic, social and pandemic-related factors potentially predicting psychological well-being were assessed. Factors assessed prior to the pandemic (T0) included age, sex, socioeconomic status (SES) and perceived social support. SES was assessed and operationalized as the participants’ highest education (1 = high school, 2 = apprenticeship, 3 = higher education/college). Social support was assessed with the 14-item short form of the *Social Support Questionnaire* (*F-SozU K14*). This questionnaire measures the perceived extent of support from the personal social network that is available if needed. The three dimensions *emotional support, practical support* and *social integration* were summed according to the manual. The F-Soz-U K14 has excellent internal consistency. Higher scores indicate more social support [28].

Further, several factors were assessed during the pandemic (T1): Whether participants a) were in a relationship, b) lived with children, c) perceived themselves or d) a household member at risk for a severe disease course in case of infection with SARS-Cov-2 (e.g., due to age, high blood pressure, diabetes, pulmonary or cardiac disease, etc). Further, the risk perception of the COVID-19 pandemic was assessed with three items on a 5-point Likert scale from “not at all” to “very much”: e) “I consider COVID-19 to be a serious issue”, f) “I am concerned about becoming infected” and g) “I am concerned that a family member or friend becomes infected”. At last, the perceived negative (“how stressful were changes”) and positive (“how positive were changes”) impact of the pandemic on h) social contacts and i) daily routines was assessed on a 5-point Likert scale from “not at all” to “very much”.

### Statistical Analysis

Group-level: Mean psychological well-being was compared to normative data at each measurement timepoint with Mann-Whitney-U tests, and between before the pandemic (T0) and during the pandemic (T1 to T5) with Wilcoxon tests. Individual-level: To identify non-linear trajectories in psychological well-being over time, latent variable mixture modelling was conducted with data collected across all six assessment timepoints (T0 to T5) using maximum likelihood estimation. As we were mainly interested in identifying distinct response patterns over time, rather than modelling structured trajectories, we treated individual scores at different time points as independent indicators and used latent profile analysis for this purpose. The R package “tidyLPA” was used [29]. Unconditional models with an increasing number of profile solutions up to ten profiles were compared to the following model fit indices: Lowest AIC and BIC, a significant Bootstrap Likelihood Ratio test (i.e., p-value <0.05) and entropy >0.8 [30]. Only profiles with ≥20 subjects were considered to maintain power for further analysis.

To identify potential predictors of different trajectories of psychological well-being, an ordinal regression analysis was conducted. Accordingly, the individual’s profile assignment identified by latent variable mixture modelling was included as dependent variable. Demographic, social and pandemic-related factors were considered as predictors. As the timing of the T0 assessments varied across participants, the time elapsed between T0 and T1 (M [SD] = 2.7 [1.8]) was considered as covariate but remained below significance in all models (*p* >0.1) and was thus omitted. An ordinal regression was chosen due to a clear and meaningful ordering of the degree of impairment in well-being across time. All risk factors were included into a multivariate model at once to identify independent associations between a predefined set of risk factors and low mental well-being, to account for potential confounding between risk factors, and to reduce multiple testing. Odds ratio (*OR*) and McFadden *R* ^*2*^ were reported as effect size for predictors and model fit, whereby McFadden *R* ^*2*^ between 0.2 and 0.4 indicate a good model fit [31].

Statical analyses were conducted with *R* [32]. Missing data was estimated with imputation by chained equation with 50 iterations and one imputation using the package *“mice”* in *R* [33]. *P*-values below 0.05 were considered statistically significant.

## Results

### Mean Well-Being Compared to Normative Data (Group-Level)

**FIGURE 1:**
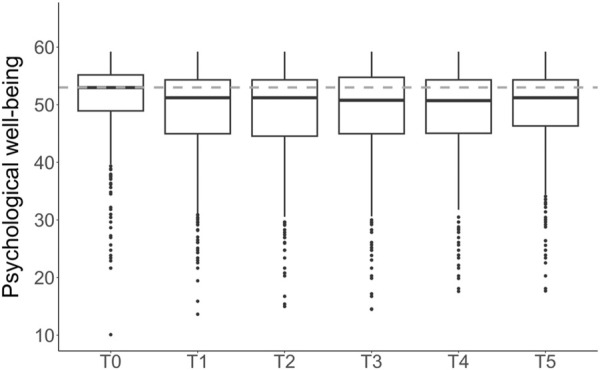
Psychological well-being at group-level prior to the outbreak of the COVID-19 pandemic (T0) and over the course of 2 years (T1 to T5) (Zurich, Switzerland. 2025). Note. Higher scores indicate better psychological well-being. The box represents the interquartile range. The thick line within the box corresponds to the median. Dots represent outliers. The dashed line shows the normative median (T = 53; [27]). T0 = before the outbreak of the pandemic. T1 = April/May 2020. T2 = October/November 2020. T3 = April/May 2021. T4 = October/November 2021. T5 = April/May 2022.

A total of 481 individuals (64% female) between 18 and 74 years of age (at T0) participated in this study. Overall sample descriptives are reported in Table 1 and sample descriptives stratified by study are reported in Supplementary Table S2. Of 481 participants, 212 (44%) participated at each time point, 155 (32%) participated at four or five time points, and 114 (23%) participated at two or three time points. Overall, 21% of data was missing. Sample descriptives are compared between those with and without missing data points in Supplementary Table S3. At group-level, comparison with norms revealed significantly lower mean psychological well-being at T0 with small effect size and significantly lower mean psychological well-being at T1 to T5 with moderate to large effect size (see Figure 1; Table 2). In comparison to before the pandemic (T0), mean psychological well-being was significantly lower at each time-point during the pandemic on a group-level (Table 3; Figure 1). Psychological well-being during the pandemic did not differ significantly between timepoints (T1 to T5, all *p* >0.30).

**TABLE 1:** Sample descriptives (n = 481) (Zurich, Switzerland. 2025).

Descriptives	Estimate	Missing data (*N*)
Female sex, *N (%)*	310 (64%)	0
Age (years) at T0, *M (SD)*	41.3 (14.0)	0
Age (years) at T1, *M (SD)* ^a^	43.8 (12.7)	0
Highest education, *N (%)*		13
High School	14 (3%)	
Apprenticeship	210 (45%)	
Higher education/college	244 (52%)	
Participant perceived themselves at risk for a severe disease course in case of having COVID-19, *N (%)*	171 (38%)	32
Participant perceived a household member at risk for a severe disease course in case of having COVID-19, *N (%)*	160 (36%)	34
Living in a relationship, *N (%)*	356 (78%)	25
Caring for children living in the same household, *N (%)*	246 (51%)	24

Note. The current study sample consists of 5 original studies (Study 1; young adults with congenital heart disease n = 101, healthy young adults n = 48; Study 2: parents of very preterm born children n = 54, parents of term-born children n = 71; Study 3: parents of children with congenital heart disease n = 99; Study 4: healthy adults n = 83; Study 5: healty adults who were term born n = 8 or preterm born n = 17.

^a^ Time difference between T0 and T1: M (SD) = 2.7 (1.8). M: Mean. SD: Standard deviation.

**TABLE 2:** Psychological well-being at group-level compared to normative data and before vs. during the pandemic (Zurich, Switzerland. 2025).

			Compared to normative mean		Compared to mean at T0	
Time points	Median (IQR)	Pseudo Median (CI-95)^a^	P-value	Effect size^b^	P-value	Effect size^b^
T0	53.0 (48.9–55.2)	52.3 (51.6–52.7)	<0.001	0.16	-	-
T1	51.2 (45.0–54.3)	49.9 (49.2–50.5)	<0.001	0.60	<0.001	0.28
T2	51.2 (44.6–54.3)	49.6 (48.8–50.4)	<0.001	0.49	<0.001	0.36
T3	50.8 (45.0–54.8)	49.9 (49.0–50.6)	<0.001	0.45	<0.001	0.35
T4	50.7 (45.1–54.3)	49.6 (48.7–50.5)	<0.001	0.50	<0.001	0.35
T5	51.2 (46.3–54.3)	50.2 (49.2–51.0)	<0.001	0.43	<0.001	0.29

Note: Higher scores indicate better psychological well-being. Mann-Whitney-U test comparing the median of the study sample to the normative pseudo median (i.e., reference value) = 53 [27].

^a^ Pseudo median considers skewness of data (Hollander & Wolfe, 1973).

^b^ Pearson correlation according to Fritz, Morris, & Richler, 2012. 0.1 = small effect, 0.3 = moderate effect, 0.5 = large effect. T0 = before the outbreak of the pandemic. T1 = April/May 2020. T2 = October/November 2020. T3 = April/May 2021. T4 = October/November 2021. T5 = April/May 2022.

**TABLE 3:** Fit indices of latent variable mixture modelling (Zurich, Switzerland. 2025).

Number of profiles	AIC	BIC	Entropy	Smallest profile size (*n*; %)	p-value^a^
1	20,498.65	20,548.76	1	481 (100%)	
2	19,749.38	19,828.72	0.93	77 (16%)	0.01
**3**	**19,575.44**	**19,684.01**	**0.88**	**25 (5%)**	**0.01**
4	19,457.06	19,594.87	0.87	10 (2%)	0.01
5	19,277.63	19,444.66	0.95	10 (2%)	0.01
6	19,230.77	19,427.04	0.8	10 (2%)	0.01
7	19,212.03	19,437.53	0.82	10 (2%)	0.01
8	19,224.78	19,479.51	0.7	10 (2%)	0.88
9	19,106.01	19,389.97	0.83	10 (2%)	0.01
10	19,079.44	19,392.63	0.84	10 (2%)	0.01

Note. Latent profile analysis conducted with tidyLPA. Best fitting model displayed in bold.

^a^ Bootstrap Likelihood Ratio test compares the model fit of a profile to the next lower number profile.

### Individual Trajectories of Psychological Well-Being Over Time (Individual-Level)

**FIGURE 2:**
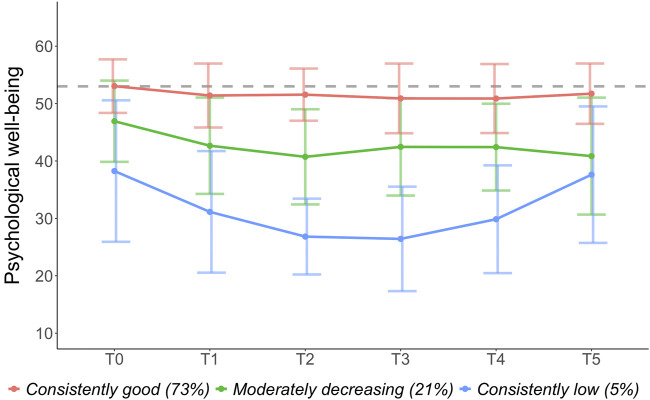
Trajectories of psychological well-being over time (Zurich, Switzerland. 2025). Note. Colored horizontal lines = mean trajectory. Vertical lines = standard error. Higher scores indicate better psychological well-being. The dashed line shows the normative median (T = 53; [27]). T0 = before the outbreak of the pandemic. T1 = April/May 2020. T2 = October/November 2020. T3 = April/May 2021. T4 = October/November 2021. T5 = April/May 2022.

The fit indices of each profile solution are reported in Table 3. The 3-profile solution met all pre-defined model fit criteria (see Section *Statistical Analysis*). The 4-, 5-, and 6-profile solutions met all model fit criteria except the smallest profile included only 10 individuals. To ensure stability of results and maintain power for further analysis, the 3-profile solution was therefore chosen, with the smallest profile group including 25 individuals. The 3-profile solution had a good entropy of 0.88, a significant Bootstrap Likelihood Ratio test (*p* < 0.01), and a smaller BIC and AIC compared to the 2-profile solution. The patterns observed in the 3-profile solution are thus considered as meaningful. The trajectory of profile 1 was labelled “consistently low psychological well-being” (*n* = 25, 5%) and was characterized by a consistently very low psychological well-being with a u-shaped trajectory with some improvement at T5. The trajectory of profile 2 was labelled “moderately decreasing psychological well-being” (*n* = 103, 21%) and was characterized by relatively low psychological well-being and a moderate decrease over time. The trajectory of profile 3 was labelled “consistently good psychological well-being” (n = 353, 73%) and was characterized by good psychological well-being across all time-points. Average trajectories per profile are displayed in Figure 2 **.** The profiles of the 6-, 5-, and 4-profile solutions are presented in Supplementary Figures S2–S4 for reference. The imputed dataset used for latent variable mixture modelling was validated by generating five and twenty additional imputed datasets using different seed values and averaging them. The averaged imputed datasets yielded similar fit indices, with the 3-profile solution meeting all predefined model fit criteria (see Supplementary Table S4 for comparison).

Ordinal regression was conducted with profile assignment as dependent variable: 3 = “consistently good psychological well-being” profile. 2 = “moderately decreasing” profile. 1 = “consistently low psychological well-being” profile. The following predictors were associated with a less favourable profile: younger age, less social support, caring for children, being concerned about someone else becoming infected with COVID-19, and perceiving changes in daily routine as stressful. Model fit was moderate to good (McFadden *R* ^*2*^ = 0.14). Statistical estimates are presented in Table 4. Post hoc, a model excluding the factor “caring for children” was estimated as females were overrepresented in this group (212 mothers, 65 fathers). When the factor “caring for children” was excluded, female sex was significantly associated with lower psychosocial well-being. All other effects remained similar (see Supplementary Table S5).

**TABLE 4:** Ordinal regression predicting profile assignment (Zurich, Switzerland. 2025).

Predictors	Odds ratio (95%-CI)	p-value
Sex (male)	1.55 (0.94–2.59)	0.089
Age	1.05 (1.03–1.08)	**<0.001**
Socioeconomic status	0.73 (0.47–1.12)	0.149
Social support	2.94 (1.97–4.44)	**<0.001**
Being in a relationship (yes)	1.17 (0.65–2.09)	0.593
Caring for children (yes)	0.51 (0.28–0.92)	**0.024**
Participant perceived themselves to be at risk for a severe COVID-19 disease course (yes)	0.69 (0.39–1.23)	0.208
Participant perceived a household member to be at risk for a severe COVID-19 disease course (yes)	1.23 (0.76–2.03)	0.404
Considers COVID-19 to be a serious issue	1.25 (0.96–1.62)	0.092
Concerned about becoming infected with COVID-19	0.92 (0.72–1.18)	0.524
Concerned about someone else becoming infected with COVID-19	0.60 (0.46–0.77)	**<0.001**
Perceived change in social contact as stressful	0.99 (0.78–1.26)	0.941
Perceived change in social contact as positive	0.85 0.66–1.11)	0.244
Perceived change in daily routine as stressful	0.72 (0.57–0.91)	**0.005**
Perceived change in daily routine as positive	1.07 (0.85–1.35)	0.539

Note. Comparison level of predictors is displayed in parentheses. Significant p-values displayed in bold. Ordinal classification of profiles: 3 = “consistently good psychological well-being” profile. 2 = “moderately decreasing” profile. 1 = “consistently low psychological well-being” profile.

## Discussion

This study investigates individual trajectories of psychological well-being in a large heterogenous group of adults from before the COVID-19 pandemic outbreak and over the subsequent 2 years. At group-level, mean psychological well-being dropped significantly during the pandemic and remained lower over the course of 2 years compared to before its outbreak. However, the inter-individual variability was high, and we identified three distinct subgroups including a subgroup with consistently good well-being, one with decreasing well-being over time, and one with consistently low well-being. Poorer psychological well-being was associate with younger age, less social support, caring for children, being concerned about someone else becoming infected with COVID-19, and perceiving pandemic-related changes in daily routine as stressful.

### Mean Psychological Well-Being Before and During the Pandemic (Group-Level)

Before the pandemic, the cohort’s psychological well-being was on average significantly below the norm with a small effect size. This suggests that the investigated sample may not be representative of the general population but includes a higher proportion of individuals at risk for compromised psychological well-being. Importantly, the cohort’s initial below-the-norm psychological well-being declined further during the pandemic and remained lower than the norm over the course of 2 years with moderate to strong effect size. These findings are partially in line with previous meta-analyses in healthy adults and vulnerable populations: A decline in psychological well-being, including increases in depressive, anxiety, and stress symptoms, has been reported, particularly during the initial phase of the pandemic [3, 4, 34–36]. In contrast, previous findings on the long-term effects have been inconsistent with substantial heterogeneity between and within populations [4, 34, 37, 38], presumably at least partly due to methodological differences between studies (e.g., national differences due to different restriction measures, different study populations), and high risk of bias due to lack of longitudinal data and/or pre-pandemic data.

### Individual Trajectories of Psychological Well-Being Throughout the Pandemic (Individual-Level)

To better understand the large variability in psychological outcomes during the COVID-19 pandemic, we conducted latent variable mixture modelling to model individual trajectories of psychological well-being and to identify distinct trajectory subgroups from before the outbreak of the pandemic and over its course until Mai 2022. Of all individuals, 73% reported on average good psychological well-being from before and throughout the pandemic; 21% reported on average lower psychological well-being before the outbreak of the pandemic, with further declines observed throughout; and 5% reported on average consistently low psychological well-being with a u-shaped trajectory of lowest psychological well-being between October 2020 and May 2021.

Only few previous studies have investigated different psychological well-being trajectories from before the outbreak of the pandemic and during 2020. These studies found that the majority of individuals (74%–90%) reported normal psychological well-being during this initial phase of the pandemic while a smaller group reported decreasing well-being over time [6, 39, 40]. Some studies further identified subgroups with recovering well-being after an initial shock, and/or consistently low well-being [6, 40]. These previous findings are largely in line with the results of the current study that expands the length of the follow-up well beyond the initial phase and 2 years into the pandemic.

Two large-scale studies investigated psychological well-being until mid-2021 in the general population but lacked pre-pandemic data: One study from Switzerland exploring depressive symptoms of more than 6000 individuals identified a large subgroup of individuals without depressive symptoms, and smaller subgroups of moderately affected or highly affected individuals [16]. Another study from Argentina in over 800 individuals identified a large subgroup of individuals without psychological distress alongside smaller subgroups with fast or slow recovery, or with increasing psychological distress throughout the pandemic [41].

Our study expands these previous findings by investigating changes in psychological well-being through mid-2022, offering additional insights into the long-term effects during a period when COVID-19 vaccines were introduced, and restriction measures were lifted. Importantly, our findings show that individuals with compromised well-being did not recover until 2 years after the outbreak of the pandemic, highlighting the profound and lasting impact this pandemic had on some individuals. Moreover, our pre-pandemic data suggest that those with lower well-being before the pandemic were more vulnerable to persistently compromised, or even worsening well-being during the pandemic.

### Risk Factors for Compromised Psychological Well-Being During the Pandemic

To better characterize the trajectory subgroups that were identified in this study, we investigated risk factors associated with a less favourable trajectory (i.e., low psychological well-being with further decline or persistently very low psychological well-being). We found that younger age, less social support, caring for children, being concerned about someone becoming infected with COVID-19, and perceiving changes in daily routine as stressful was significantly associated with a less favourable trajectory and consequently with lower psychological well-being during the pandemic.

The finding that older individuals were less likely to report low psychological well-being is in line with recent menta-analyses [38, 42–44] and illustrates a certain degree of resilience in the older population during the pandemic. Indeed, one meta-analysis found that in older adults, positive mental health indicators (e.g., psychological functioning, resilience, social support and wellbeing) mildly decreased initially but recovered and even increased during the pandemic [45]. This is particularly interesting, as older individuals were considered at higher risk for a severe disease course in the case of a SARS-CoV-2 infection [46, 47], which one might expect to affect mental health negatively. However, our analysis showed that individuals who perceived themselves at risk for a severe disease course were not more likely to report lower psychological well-being, indicating that other factors may play a more prominent role and/or that these individuals were able to successfully mobilize resources to preserve their well-being.

Our data further showed that individuals caring for children were more likely to report lower psychological well-being. This aligns with other studies indicating that parents had higher rates of anxiety and depression during the pandemic compared to adults without caregiving responsibilities [48, 49]. One explanation for this effect may be the additional responsibilities and burdens faced by parents, including the balance of home schooling, care duties and work during the pandemic [50]. This may have led to an increase in parenting stress (i.e., a response when the demands of parenting responsibilities are inconsistent with parent’s expectations or resources), which in turn, is negatively associated with psychological well-being [51].

Furthermore, we could show that less social support is linked to lower psychological well-being during the pandemic, which is in line with evidence from a systematic review on mental health during the pandemic in Europe [44]. In contrast to a broad body of research showing that females’ psychological well-being was more affected during the pandemic than males’ [35, 42–44], initially, we did not find significant sex differences in our study. This is likely due to an overrepresentation of females caring for children in our sample. In fact, female sex became a significant predictor for lower well-being when the factor caring for children was excluded from the model.

While these demographic and social risk factors may not be specific to the COVID-19 pandemic and have been associated with lower psychological well-being in other contexts [52, 53]), we identified additional risk factors that may be unique to the pandemic. We showed that being concerned about someone else becoming infected with COVID-19 was significantly associated with lower psychological well-being, while concerns about becoming infected yourself was not associated with psychological well-being. In addition, perceiving changes in daily routine during the initial phase of the pandemic (i.e., Mai/April 2020) as stressful was significantly associated with lower psychological well-being. Consistent with this, Landi and colleagues (2022) found that reduced psychological flexibility — defined as the ability to adaptively alter internal experiences in response to negative thoughts, feelings, or events [54] — was linked to higher levels of anxiety and depressive symptoms during the pandemic [55]. Taken together, individuals who perceive sudden pandemic-related changes as stressful may exhibit less flexibility in managing internal experiences, potentially leading to impaired psychological well-being.

### Implications

The COVID-19 pandemic can be considered as a multidimensional and universal stressor that has been experienced by people globally [56]. Our results imply that some individuals were more vulnerable than others during the pandemic. Specifically, parents were particularly vulnerable to experiencing compromised psychological well-being, likely due to the need to juggle multiple responsibilities, including home-schooling, caregiving, and work. Preventive and interventional support should be directed towards parents and young adults in particular. In addition, lack of social support is a well-known risk factor for poor psychological well-being and mental health problems [53, 57]. As an intervention strategy, the absence of an individual’s social network could be supplemented by professional support, e.g., from social workers. Therefore, strengthening these services beyond the pandemic may help prevent long-term mental health consequences for those at risk. At last, enhancing psychological flexibility to respond adequately to changes in daily routines could be a valuable target for public health interventions using evidence-based therapeutic strategies [55, 58].

Given the global scale of the pandemic, even a small proportion of individuals with poor psychological well-being represents millions of people experiencing mental health difficulties. Consequently, this global mental health burden has pushed many mental health services to their limits. Yet, on average, only 2% of governmental health budgets is allocated to mental health [59]. The current findings of long-lasting mental health burden of this pandemic echo call for action to prioritize mental health on political agendas.

### Strengths and Limitations

This study pooled data from pre-existing cohort studies using similar questionnaires conducted prior to the outbreak of the COVID-19 pandemic and supplemented this data with 5 follow-ups during the pandemic from April 2020 to Mai 2022. This resulted in a unique dataset on psychological well-being in a diverse sample of 481 adults, aged 18–74 years, spanning the period prior to the pandemic, the initial phase with extensive restriction measures in place, the introduction of COVID-19 vaccines, and the subsequent easing of restriction measures. Thanks to this well-powered and diverse sample we were able to investigate different trajectories of psychological well-being using latent variable mixture modelling and investigate a large set of risk factors associated with poor well-being. However, some limitations need to be considered when interpreting the results: This study used pre-pandemic data from five different prospective cohort studies, which were not initially designed for pooling or examining well-being during the pandemic. Nevertheless, all studies were conducted by the same research group and employed similar questionnaires, facilitating the data pooling process. The pre-pandemic data (T0) were collected between 2014 and 2020. Importantly, we demonstrated that the time interval between T0 and the first COVID-19 assessment (T1) was not associated with changes in psychological well-being during the pandemic. Further, the pooled study sample originates from different clinical and healthy cohorts including healthy adults with and without children, parents of children with complex medical conditions, and young adults with congenital heart disease. Thus, the study sample is not representative for the general population, but the large and diverse study sample allows for investigation of risk factors associated with psychological well-being. We did not statistically examine whether the presence of a chronic condition (e.g., congenital heart disease) was associated with psychological well-being, as the presence of chronic conditions was not consistently assessed across samples. However, we demonstrated that concerns about becoming infected with SARS-CoV-2 – likely linked to an increased risk of a severe disease course, e.g., due to the presence of a chronic condition – were not significantly associated with psychological well-being. The current study assessed psychological well-being with the self-reported mental scale of the SF-12 questionnaire which assesses health-related quality of life. We did not assess any proxy reports, and we did not assess psychiatric symptoms to inform about the prevalence of mental disorders. Finally, in this longitudinal study, 44% of participants contributed data at each timepoint, and overall, 21% of the data were missing. Missing data were imputed with MICE, but uncertainty of predicted missing values was not considered as the corresponding MICE function “pool()” was not compatible with latent class estimation using “tidyLPA.”

### Conclusion

This study in a diverse adult sample used pre-pandemic data, supplemented by 5 measurement timepoints during the pandemic until mid-2022, to examine pandemic-related changes in psychological well-being. We contribute to the extensive research on mental health during the pandemic, revealing that despite many individuals who remained resilient and reported preserved well-being, two vulnerable subgroups were identified: one experienced decreasing well-being, and another reported consistently low well-being over the course of 2 years. The pandemic affected those most who were younger, cared for children, lacked social support, were concerned about someone else becoming infected with COVID-19, and were stressed about changes in daily routine. Considering the global impact of the COVID-19 pandemic, even a small percentage of individuals in the population who are affected translates into millions of people experiencing compromised mental health. Public health measures are needed to identify and support those individuals at highest risk for mental health problems in the post-pandemic era.
