# Supplementary material for: Psychological Well-Being in Adults Across the COVID-19 Pandemic: A Two-Year Longitudinal Study
Source: Int J Public Health. 2025 Jul 25;70:1608347. doi: 10.3389/ijph.2025.1608347 (PMC12331532; doi:10.3389/ijph.2025.1608347)
Supplement: Supplementary file 1 [file DataSheet1.docx]

Supplementary Data

**Supplementary Table 1:** Overview of nationwide measures to reduce the spread of COVID-19 in Switzerland.

| **Wave** | **Nationwide restrictions** |
| --- | --- |
| T1  (April/May 2020) | School closure |
|  | Prohibition of public events |
|  | Prohibition of private events |
|  | Closure of nonessential and retail services |
|  | Reduction of public transport service |
| T2  (October/November 2020) | School closure for adult education only |
|  | Mandatory face masks in public space for people at the age of 12 years or older |
|  | Prohibition of public events with >50 people |
|  | Prohibition of private events with >10 people |
|  | Prohibition of leisure activities with >15 people |
|  | Restriction in restaurants to 4 people per table and earlier closing times |
| T3  (April/May 2021) | Prohibition of adult education with > 50 people |
|  | Mandatory face masks in public space for people at the age of 12 years or older |
|  | Prohibition of public events with >50 people (inside) and >100 people (outside) |
|  | Prohibition of private events with >15 people |
|  | Prohibition of leisure activities with >15 people |
|  | Inner area of restaurants closed; outdoor area restricted to 4 people per table |
| T4  (October/November 2021) | Adult education with COVID certificate |
|  | Mandatory face masks in public indoor space for people at the age of 12 years or older (no mandatory face masks when COVID certificate is required) |
|  | COVID certificate for all public events inside (outside only with > 500 people) |
|  | Private events restricted to <30 people inside (outside <50 people) |
|  | No restrictions for leisure activities with <30 people. COVID certificate for leisure activities with >30 people. |
|  | Inner area of restaurants, bars and clubs is open for people with COVID certificate |
| T5  (April/Mai 2022) | No restrictions |

*Note*. Some cantons may have ordered stricter measures. Source: Federal Office of Public Health (FOPH) Switzerland.

**Supplementary Figure 1:** Recruitment flow chart

**
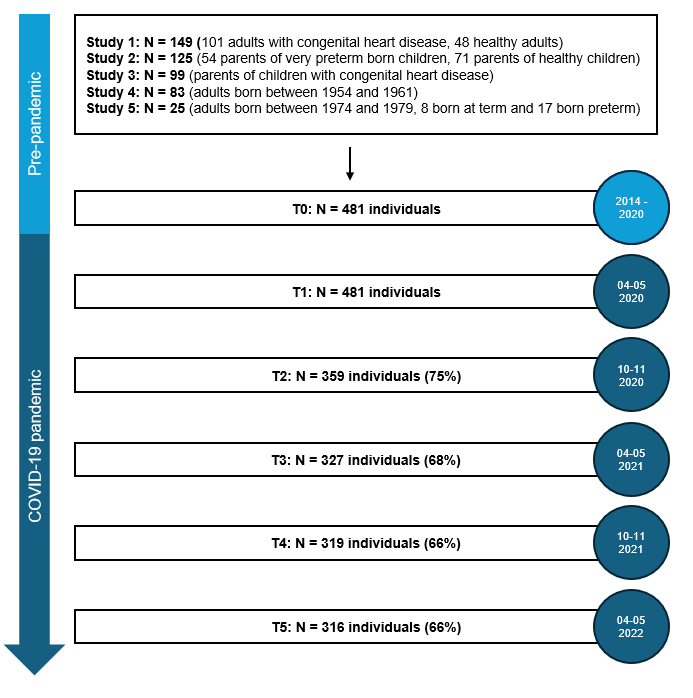
**

***Supplementary Table 2: Sample descriptives stratified by study***

| Descriptives | Study 1 (n = 149) | Study 2 (n = 125) | Study 3 (n = 99) | Study 4 (n = 83) | Study 5 (n = 25) |
| --- | --- | --- | --- | --- | --- |
| Recruitment and sample size | *Young adults with congenital heart disease n = 101;*  *Healthy young adults n = 48* | *Parents of very preterm born children n = 54;*  *Parents of term-born children n = 71* | *Parents of children with congenital heart disease n = 99* | *Healthy adults n = 83* | *Healthy adults who were*  *term born n = 8 or preterm born n = 17* |
|  |  |  |  |  |  |
| Female sex, *N (%)* | 64 (43%) | 116 (93%) | 69 (70%) | 45 (54%) | 16 (64%) |
| Age (years) at T0, *M (SD)* | 25.2 (3.8) | 43.1 (5.3) | 43.1 (5.2) | 64.8 (1.2) | 43.4 (1.6) |
| Age (years) at T1, *M (SD)*^a^ | 29.7 (3.8) | 44.0 (5.3) | 46.6 (5.5) | 65.3 (1.2) | 44.0 (1.5) |
| Highest education, *N (%)* |  |  |  |  |  |
| High School | 11 (7%) | 1 (1%) | 1 (1%) | 1 (1%) | 0 (0%) |
| Apprenticeship | 93 (62%) | 29 (23%) | 48 (48%) | 35 (42%) | 5 (20%) |
| Higher education/college | 44 (30%) | 91 (73%) | 42 (42%) | 47 (57%) | 20 (80%) |
| Participant perceived themselves at risk for a severe disease course in case of having COVID-19, *N (%)* | 85 (57%) | 7 (6%) | 13 (13%) | 66 (80%) | 0 (0%) |
| Participant perceived a household member at risk for a severe disease course in case of having COVID-19, *N (%)* | 34 (23%) | 27 (22%) | 60 (60%) | 36 (43%) | 3 (12%) |
| Living in a relationship, *N (%)* | 94 (63%) | 90 (72%) | 92 (93%) | 58 (70%) | 22 (88%) |
| Caring for children living in the same household, *N (%)* | 31 (21%) | 100 (80%) | 93 (94%) | 4 (5%) | 18 (72%) |

*Note. Missing data: Highest education n = 13, themselves at risk for a severe disease course n = 32, a household member at risk for a severe disease course n = 34, Living in a relationship n = 25, Caring for children n = 24.*

***Supplementary Table 3: Comparison of participants with and without missing data points***

| Descriptives | Participated at each follow up / no missing data points | One or two missing data points | Comparison no vs. one or two missing data points  ***p*-value** | Three or four missing data points | Comparison no vs. three or four missing data points  ***p*-value** |
| --- | --- | --- | --- | --- | --- |
| Sample size | 212 (44%) | 155 (32%) |  | 114 (23%) |  |
|  |  |  |  |  |  |
| Female sex, *N (%)* | 142 (67%%) | 104 (67%) | 1.0 ^+^ | 64 (56%) | 0.070 ^+^ |
| Age (years) at T0, *M (SD)* | 42.9 (15.2) | 41.4 (12.7) | 0.307 ° | 38.2 (12.8) | **0.003 °** |
| Age (years) at T1, *M (SD)*^a^ | 45.1 (13.8) | 43.9 (11.7) | 0.386 ° | 41.1 (11.6) | **0.006 °** |
| Highest education, *N (%)* |  |  |  |  |  |
| High School | 5 (2%) | 3 (2%) |  | 6 (5%) |  |
| Apprenticeship | 90 (42%) | 64 (41%) |  | 56 (49%) |  |
| Higher education/college | 114 (54%) | 82 (53%) | 0.669 ^#^ | 48 (42%) | **0.034** ^#^ |
| Participant perceived themselves at risk for a severe disease course in case of having COVID-19, *N (%)* | 90 (42%) | 45 (39%) | **0.021** ^+^ | 36 (32%) | 0.197 ^+^ |
| Participant perceived a household member at risk for a severe disease course in case of having COVID-19, *N (%)* | 72 (34%) | 56 (36%) | 0.580 ^+^ | 32 (28%) | 0.540 ^+^ |
| Living in a relationship, *N (%)* | 157 (74%) | 119 (77%) | 0.306 ^+^ | 80 (70%) | 0.771 ^+^ |
| Caring for children living in the same household, *N (%)* | 95 (45%) | 90 (78%) | **0.005** ^+^ | 61 (54%) | 0.095 ^+^ |

*Note. Missing data: Highest education n = 13, themselves at risk for a severe disease course n = 32, a household member at risk for a severe disease course n = 34, Living in a relationship n = 25, Caring for children n = 24.* Test statistics: + two-sampled t-test, ° Wilcoxon rank sum test, ^#^ Chi-square test

**Supplementary Figure 2**: LPA 6-profile solution


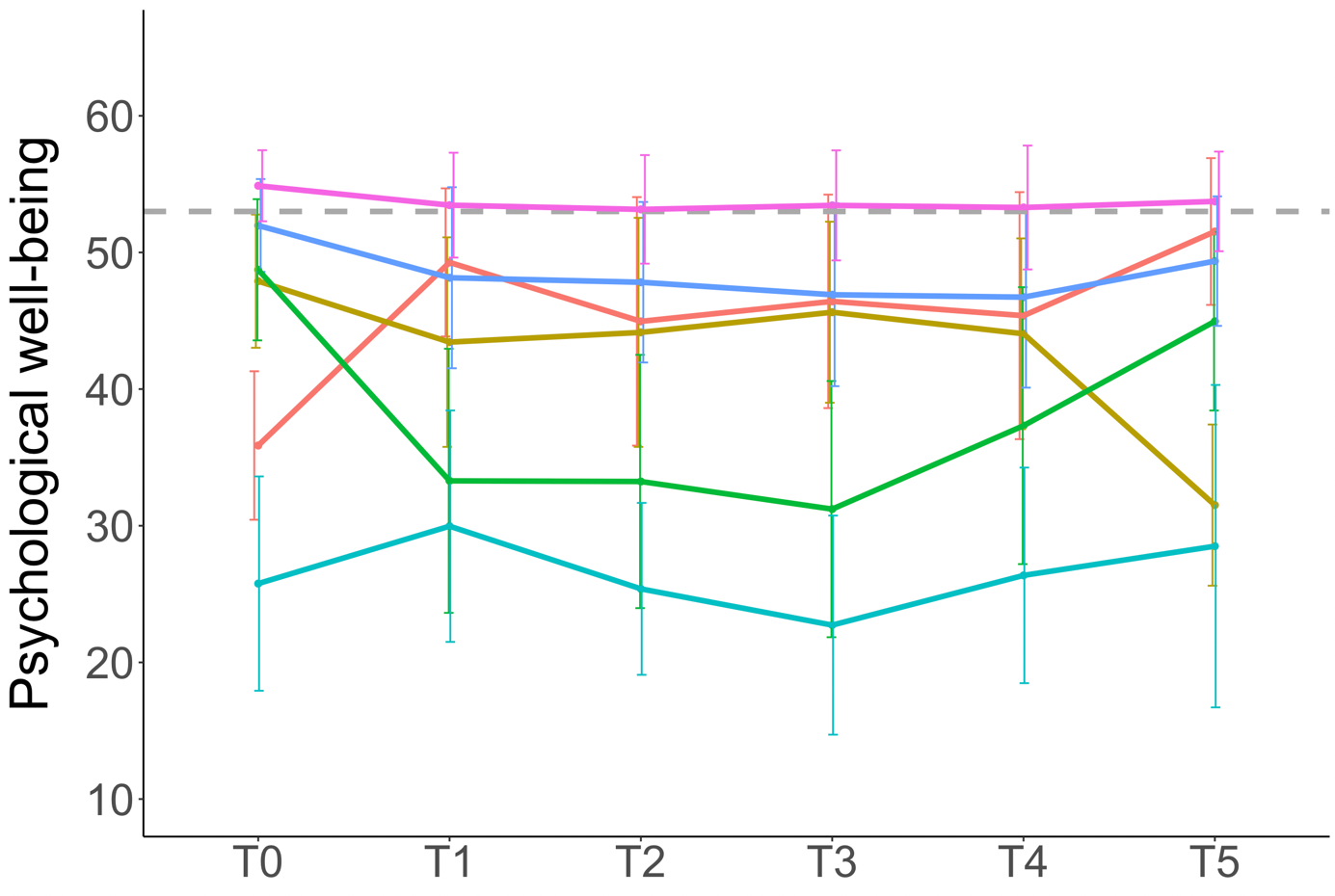


*Note. Colored horizontal lines = mean trajectory. Vertical lines = standard error. Higher scores indicate better psychological well-being. The dashed line shows the normative median (T = 53; Wirtz et al., 2018). T0 = before the outbreak of the pandemic. T1 = April/May 2020. T2 = October/November 2020. T3 = April/May 2021. T4 = October/November 2021. T5 = April/May 2022.*

**Supplementary Figure 3**: LPA 5-profile solution


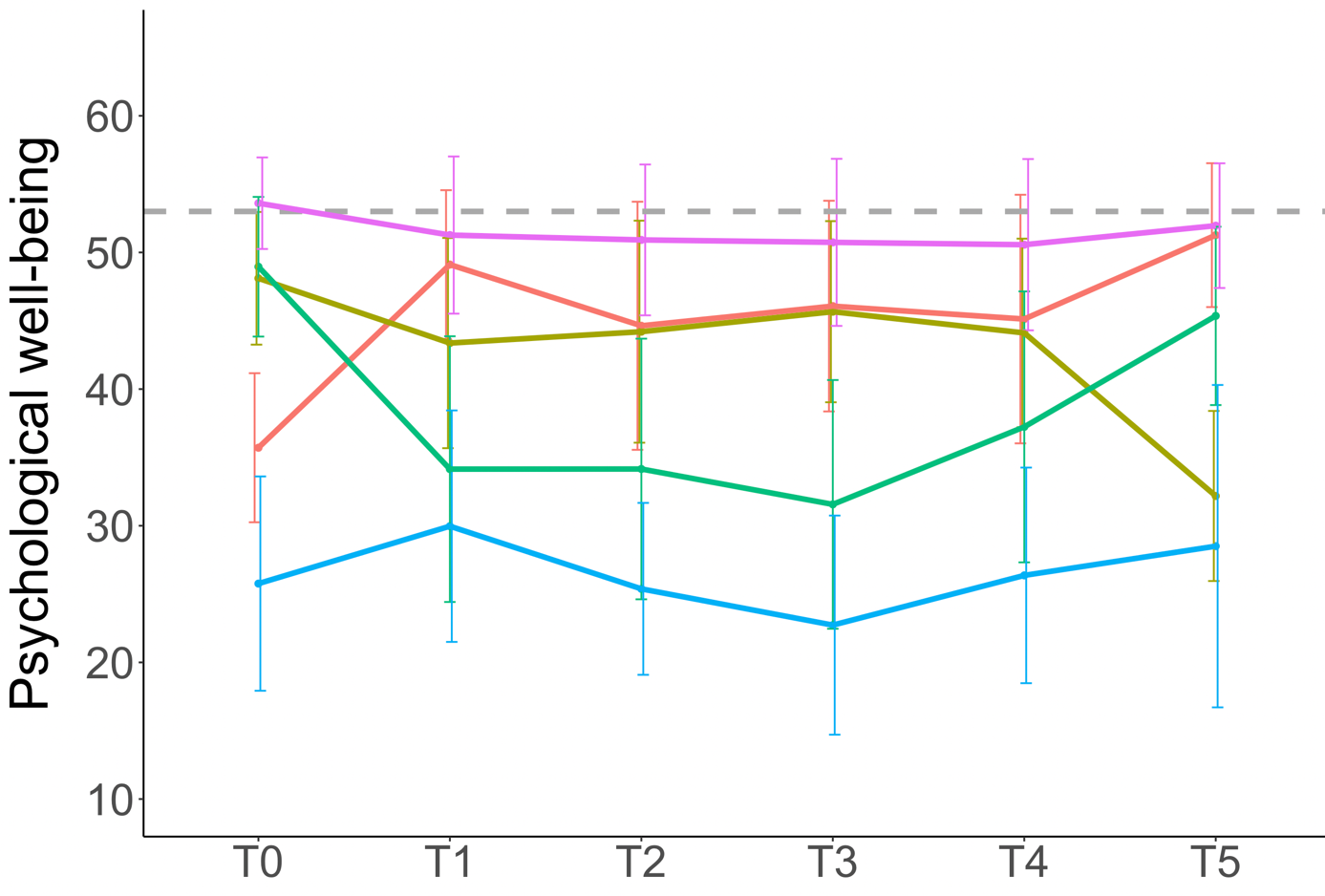


*Note. Colored horizontal lines = mean trajectory. Vertical lines = standard error. Higher scores indicate better psychological well-being. The dashed line shows the normative median (T = 53; Wirtz et al., 2018). T0 = before the outbreak of the pandemic. T1 = April/May 2020. T2 = October/November 2020. T3 = April/May 2021. T4 = October/November 2021. T5 = April/May 2022.*

**Supplementary Figure 4**: LPA 4-profile solution


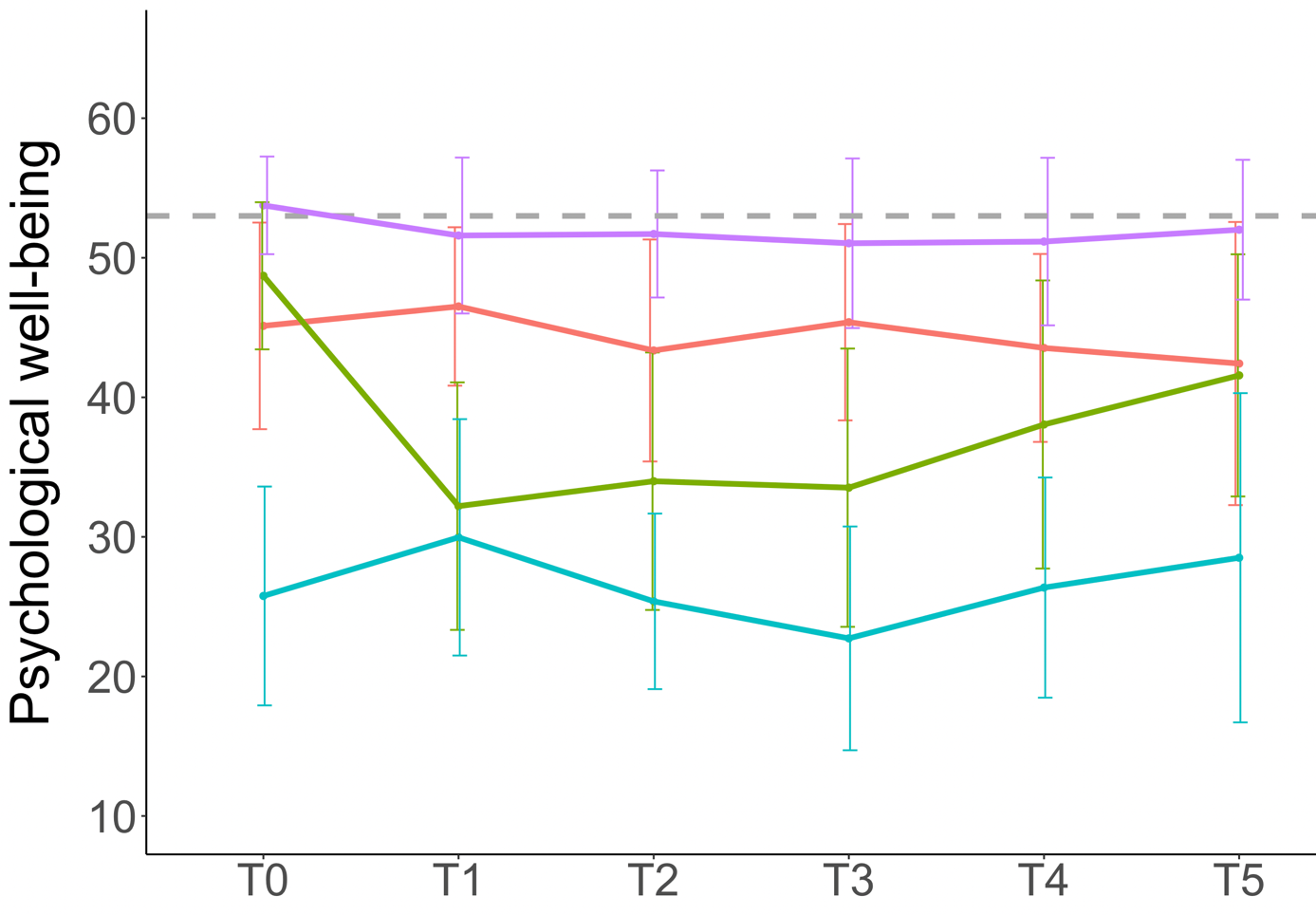


*Note. Colored horizontal lines = mean trajectory. Vertical lines = standard error. Higher scores indicate better psychological well-being. The dashed line shows the normative median (T = 53; Wirtz et al., 2018). T0 = before the outbreak of the pandemic. T1 = April/May 2020. T2 = October/November 2020. T3 = April/May 2021. T4 = October/November 2021. T5 = April/May 2022.*

**Supplementary Table 4: Validation of the imputed dataset based on the average of five imputations using different seed values. Fit indices of latent variable mixture modelling**

| **5 imputations (averaged)** | | | | | |
| --- | --- | --- | --- | --- | --- |
| Number of profiles | AIC | BIC | Entropy | Smallest profile size (*n*; %) | p-value^a^ |
| 1 | 20127.26 | 20177.37 | 1 | 481 (100%) |  |
| 2 | 19296.61 | 19375.95 | 0.93 | 82 (17%) | 0.01 |
| **3** | **19058.36** | **19166.93** | **0.85** | **34 (7%)** | **0.01** |
| 4 | 18952.88 | 19090.68 | 0.87 | 10 (2%) | 0.01 |
| 5 | 18950.86 | 19117.89 | 0.9 | 14 (3%) | 0.02 |
| 6 | 18868.02 | 19064.29 | 0.83 | 14 (3%) | 0.01 |
| 7 | 18728.97 | 18954.47 | 0.86 | 10 (2%) | 0.01 |
| 8 | 18680.83 | 18935.56 | 0.86 | 10 (2%) | 0.01 |
| 9 | 18657.69 | 18941.65 | 0.86 | 10 (2%) | 0.01 |
| 10 | 18618.75 | 18931.94 | 0.87 | 5 (1%) | 0.01 |
| **20 imputations (averaged)** | | | | | |
| Number of profiles | AIC | BIC | Entropy | Smallest profile size (*n*; %) | p-value^a^ |
| 1 | 20034.02 | 20084.13 | 1 | 481 (100%) |  |
| 2 | 19164.01 | 19243.35 | 0.93 | 82 (17%) | 0.01 |
| **3** | **18924.59** | **19033.16** | **0.89** | **24 (5%)** | **0.01** |
| 4 | 18855.6 | 18993.41 | 0.76 | 19 (4%) | 0.01 |
| 5 | 18793.6 | 18960.64 | 0.79 | 14 (3%) | 0.01 |
| 6 | 18732.98 | 18929.25 | 0.81 | 14 (3%) | 0.01 |
| 7 | 18747.01 | 18972.51 | 0.74 | 1 (<1%) | 0.97 |
| 8 | 18621.84 | 18876.57 | 0.77 | 1 (<1%) | 0.01 |
| 9 | 18529.67 | 18813.63 | 0.85 | 10 (2%) | 0.01 |
| 10 | 18520.58 | 18833.77 | 0.77 | 5 (1%) | 0.02 |

*Note. Latent profile analysis conducted with tidyLPA. ^a^Bootstrap Likelihood Ratio test compares the model fit of a profile to the next lower number profile.*

**Supplementary Table 5: Ordinal regression predicting profile assignment, excluding factor ‘caring for children’**

| Predictors | Odds ratio (CI-95) | p-value |
| --- | --- | --- |
| **Sex (male)** | **1.70 (1.04 to 2.83)** | **0.033** |
| **Age** | 1.05 (1.02 to 1.07) | **<0.001** |
| SES | 0.72 (0.47 to 1.10) | 0.133 |
| **Social support** | 2.83 (1.90 to 4.25) | **<0.001** |
| Being in a relationship (yes) | 0.92 (0.55 to 1.54) | 0.746 |
| Participant perceived themselves to be at risk for a severe COVID-19 disease course (yes) | 1.18 (0.73 to 1.94) | 0.506 |
| Participant perceived a household member to be at risk for a severe COVID-19 disease course (yes) | 0.97 (0.55 to 1.67) | 0.902 |
| Considers COVID-19 to be a serious issue | 1.24 (0.96 to 1.61) | 0.101 |
| Concerned about becoming infected with COVID -19 | 0.89 (0.70 to 1.14) | 0.361 |
| **Concerned about someone else becoming infected with COVID-19** | 0.62 (0.48 to 0.80) | **<0.001** |
| Perceived change in social contact as stressful | 1.01 (0.79 to 1.28) | 0.965 |
| Perceived change in social contact as positive | 0.88 (0.67 to 1.14) | 0.321 |
| **Perceived change in daily routine as stressful** | 0.72 (0.57 to 0.90) | **0.004** |
| Perceived change in daily routine as positive | 1.06 (0.84 to 1.33) | 0.629 |

*Note*. Comparison level of predictors is displayed in parentheses. Ordinal classification of profiles: 3 = “consistently good psychological well-being” profile. 2 = “moderately decreasing” profile. 1 = “consistently low psychological well-being” profile.
